# Achieving Quality and Effectiveness in Dementia Using Crisis Teams (AQUEDUCT): a study protocol for a randomised controlled trial of a Resource Kit

**DOI:** 10.1186/s13063-021-05995-y

**Published:** 2022-01-18

**Authors:** Donna Maria Coleston-Shields, David Challis, Angela Worden, Emma Broome, Tom Dening, Boliang Guo, Juanita Hoe, Brynmor Lloyd-Evans, Esme Moniz-Cook, Steve Morris, Fiona Poland, David Prothero, Martin Orrell

**Affiliations:** 1 Mental Health & Clinical Neurosciences, University of Nottingham, Nottingham, UK; 2 NIHR Nottingham Biomedical Research Centre, Hearing Sciences, University of Nottingham, Nottingham, UK; 3 School of Medicine, University of Nottingham, Nottingham, UK; 4 School of Health Sciences, City, University of London, London, UK; 5 Division of Psychiatry, University College London, London, UK; 6 Faculty of Health Sciences, University of Hull, Hull, UK; 7 Department of Public Health and Primary Care, University of Cambridge, Cambridge, UK; 8 School of Health Sciences, University of East Anglia, Norwich, UK; 9 Institute of Mental Health, University of Nottingham, Nottingham, UK

**Keywords:** Dementia, Crisis teams, Hospitalisation, Home support, Quality of care

## Abstract

**Background:**

Improving care at home for people with dementia is a core policy goal in the dementia strategies of many European countries. A challenge to effective home support is the occurrence of crises in the care of people with dementia which arise from changes in their health and social circumstances. Improving the management of these crises may prevent hospital admissions and facilitate better and longer care at home. This trial is part of a National Institute for Health Research funded programme, AQUEDUCT, which aims to improve the quality and effectiveness of teams working to manage crises in dementia.

**Methods/design:**

It is a pragmatic randomised controlled trial of an online Resource Kit to enhance practice in teams managing crises in dementia care. Thirty teams managing mental health crises in dementia in community settings will be randomised between the Resource Kit intervention and treatment as usual. The primary outcome measure is psychiatric admissions to hospital for people with dementia in the teams’ catchment area recorded 6 months after randomisation. Other outcomes include quality of life measures for people with dementia and their carers, practitioner impact measures, acute hospital admissions and costs. To enhance understanding of the Resource Kit intervention, qualitative work will explore staff, patient and carers’ experience.

**Discussion:**

The Resource Kit intervention reflects current policy to enable home-based care for people with dementia by addressing the management of crises which threaten the viability of care at home. It is based upon a model of best practice for managing crises in dementia designed to enhance the quality of care, developed in partnership with people with dementia, carers and practitioners. If the Resource Kit is shown to be clinically and cost-effective in this study, this will enhance the probability of its incorporation into mainstream practice.

**Trial registration:**

ISRCTN 42855694; Registered on 04/03/2021; Protocol number: 127686/2020v9; Research Ethics Committee, 09/03/2021, Ref 21/WM/0004; IRAS ID: 289982

**Supplementary Information:**

The online version contains supplementary material available at 10.1186/s13063-021-05995-y.

## Background

Worldwide around 47 million people live with dementia [1] including an estimated 850,000 people in the UK [2]. Services for people with dementia in the UK cost over £17 billion a year, even with savings of around £6 billion a year due to the contribution of family caregivers. In future decades, with an ageing population, increasing numbers of people with dementia are likely to live at home. As in many countries, improving dementia care is a key priority in England [3, 4], with home-oriented care a key objective of the National Dementia Strategy [5]. However, fluctuations in the health and social circumstances of the person with dementia and their family carers may lead to crisis, breakdown in home care and admissions to hospital or long-term care. Focused approaches to manage these problems may sustain care at home, improve quality of life and reduce costs.

Indeed, in one study, one in ten respondents reported a relative with dementia admitted to hospital unnecessarily due to lack of access to community support [2]. For the working age population with mental health problems, Crisis Resolution Teams, designed to avoid inappropriate hospital admission, have shown some reduction in hospital admissions and improvements in patient and caregiver satisfaction [6–10] although requiring better-defined service models [11]. In contrast, people with dementia and their carers experiencing crisis are often supported through a variety of different services varying in nomenclature, staff mix and operational procedure [12]. These services include Community Mental Health Teams, Crisis Resolution and Home Treatment Teams and generic older people’s rapid response teams. All these services are described in this paper as Teams Managing Crises in Dementia (TMCDs). Unlike services for younger adults, there appears to be less evidence and no guidance as to how such teams, or crisis resolution services for older people including those with dementia, should be designed or operate [13, 14].

People with dementia, caregivers, and practitioners appear to value a more coordinated approach to crisis management which is responsive to the unique features of each crisis [15]. Home Treatment Packages have been used to help teams manage crises for people with dementia and their family carers [16], with specialist older people’s crisis services identified as providing valuable expertise [17, 18]. A systematic review and a scoping exercise of crisis interventions in dementia found some evidence that specialist crisis teams effectively managed crises and reduced hospital admissions, although a more clearly defined model of best practice was needed [19]. This suggested that crisis intervention approaches could reduce admissions to psychiatric hospitals for people with dementia, but that stronger evidence to support their efficacy was required and this informed the development of the AQUEDUCT research programme.

AQUEDUCT is a National Institute for Health Research (NIHR) funded programme (RP-PG-0612-20004) designed to develop and evaluate a coherent model of crisis management in dementia care, reducing service variation and enhancing quality and effectiveness. Earlier parts of the programme involved a scoping review of the effectiveness of crisis interventions and a survey of crisis teams which confirmed some evidence of impact upon hospital admissions but with marked variations across services in England in the pathways to manage crises. It recommended a trial of a standardised care pathway and measurable intervention to provide better evidence [12]. Work with people with dementia, carers and practitioners in mental health services was undertaken using qualitative methods to develop a model of best practice and associated information and training materials for TMCDs [14, 20]. This model underpins a Resource Kit, developed in the AQUEDUCT programme, designed to provide guidance on best practice and achieve high-quality care.

**Fig. 1:**
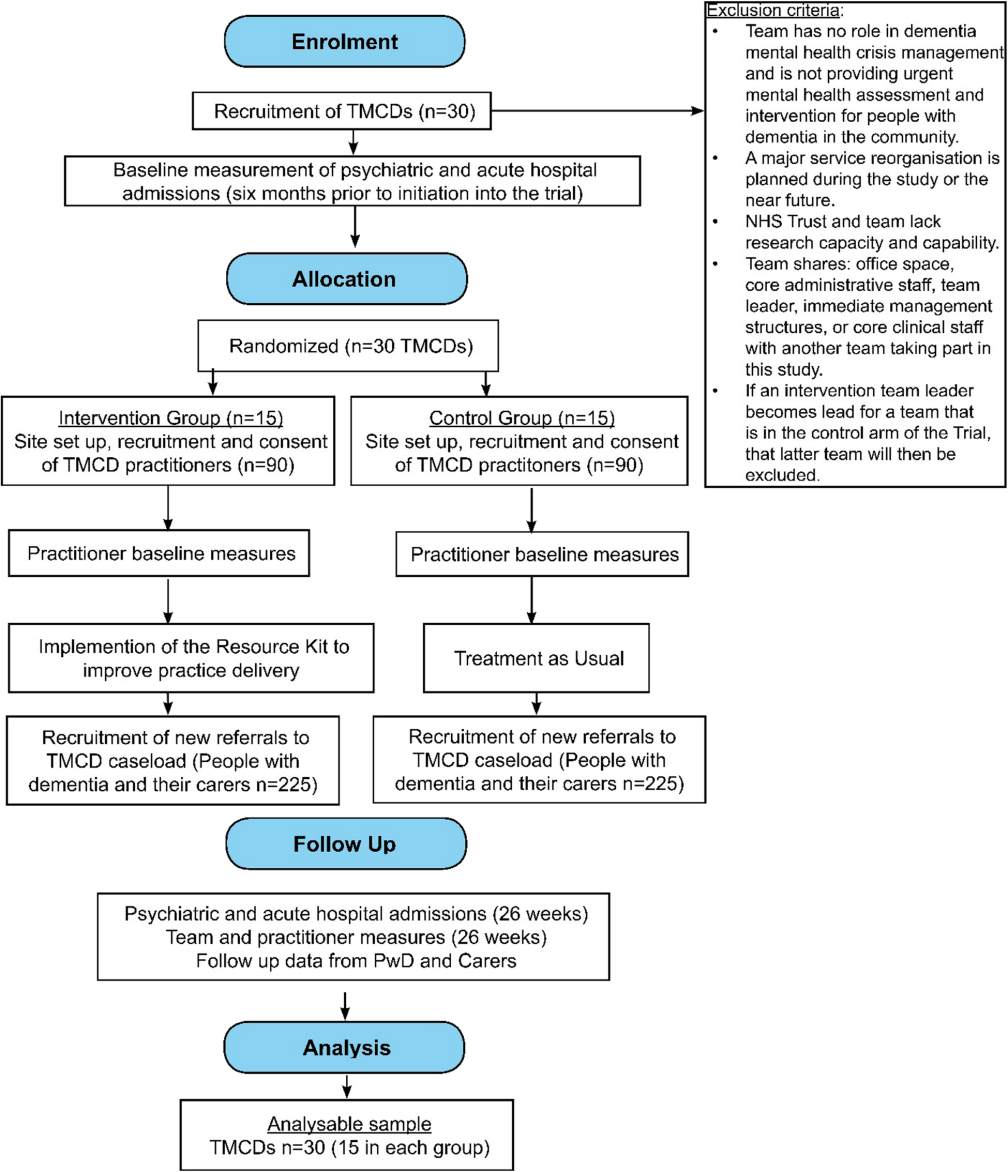
The AQUEDUCT Trial flowchart

The AQUEDUCT Trial aims to evaluate the Resource Kit in practice by conducting a randomised controlled trial with a representative sample of TMCDs across England, examining the impact upon hospital admissions, costs, and upon people with dementia, carers and staff compared with treatment as usual (TAU). A Consolidated Standards of Reporting Trials (CONSORT) flowchart of the trial is shown in Fig. 1.

## Method/design

The ‘Standard Protocol Items: Recommendation for Interventional Trials’ (SPIRIT) reporting guidelines have been used [21]—see the completed SPIRIT checklist (Additional file 1).

### Governance

Several groups have been established to oversee the trial. The Programme Management Group (PMG) oversees the full AQUEDUCT programme of research. It meets every 6 months and comprises the Chief Investigator (CI), all collaborators, researchers, and patient and public involvement (PPI) representatives. A Trial Steering Group (TSG) has met during set-up and thereafter at least annually. The TSG membership, approved by the funder, includes an independent Chair, at least two independent members with appropriate methodological and clinical expertise, one or two PPI representatives (a person with dementia or carer), the CI and other relevant members of the Trial Management Group (see below). Representatives from NIHR and from the trial sponsor (Nottinghamshire Healthcare NHS Foundation Trust) may also attend TSG meetings as active members. The role of the TSG is to: advise the CI on all aspects of the trial; provide overall supervision of the trial protocol, case report form, and statistical analysis; monitor trial progress; review relevant information from other sources related to the trial; review outputs and final reports; and if necessary, prematurely close the trial. The programme also has a standing PPI Reference Group that meets 6 monthly and is consulted regarding study development.

A Trial Management Group (TMG) has met monthly during the set-up of the trial and will then meet monthly or bimonthly, as appropriate and is accountable to the TSG for trial implementation and operation. It consists of key individuals directly involved in the development and delivery of the trial including the: CI; Trial Manager; collaborators; and experts by experience. The role of the TMG is to: monitor trial progress, ensure compliance with and adherence to the project plan; identify and resolve concerns regarding the intervention and research, including publication authorship; and consider and act on recommendations of the TSG and Research Ethics Committee.

A Data Monitoring Committee (DMC), independent of the sponsor and trial, will meet on at least two to three occasions and consists of a Chair, a statistician, and a clinical researcher. Membership of the DMC has been approved by the funding body. The role of the DMC is to: review the trial protocol and study materials regarding data management and analysis pertinent to their duties as the DMC; advise the TSG when it believes the trial protocol should be altered; and advise the TSG if the committee feels the trial should be prematurely closed [22].

The protocol for this present work package was developed in consultation with a Clinical Staff Reference Group, consisting of National Health Service (NHS) practitioners currently working in TMCDs across England, and the PPI Reference Group. All study documentation and participant recruitment procedures have been reviewed by the patient and public involvement representatives.

### Design

This is a pragmatic randomised controlled trial of an online Resource Kit for use by TMCDs. It is a two-arm, parallel-group, TAU controlled trial with treatment allocation of teams on a 1:1 ratio. The study aims to randomise 30 teams managing mental health crises in dementia in community settings between the Resource Kit intervention and treatment as usual. The null hypothesis is that there is no difference in the effect of crisis care management between teams using the Resource Kit and TAU. The primary outcome measure is admissions to psychiatric hospitals of people with dementia in the teams’ catchment area, assessed over a 6-month period after randomisation.

A feasibility study was conducted for the trial of this Resource Kit, including qualitative work to further shape the intervention by taking account of participant experiences and preferences [23]. This demonstrated that the Resource Kit was acceptable to and usable by practitioners working in TMCDs, who consistently reported that it accurately reflected their clinical practice. The feasibility study also identified certain difficulties experienced by people with dementia and their carers participating directly in research concerning their TMCD during a personal crisis. Building on these findings, iterative consultation with both the PPI Reference Group and the Clinical Staff Reference Group refined the trial of the Resource Kit in Crisis Management addressing both the content of the intervention itself and reducing the demands on participants and the scale of data collection processes. Implementation is designed to be mindful of pressures on the NHS and research feasibility due to the COVID-19 pandemic.

### Intervention

**Fig. 2:**
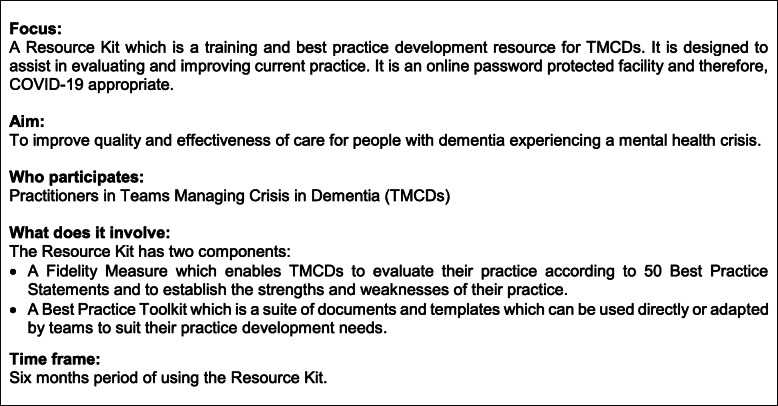
The Resource Kit intervention

The Resource Kit intervention is an online and COVID-19 appropriate resource for TMCDs consisting of two components. The first is a Fidelity Measure which enables TMCDs to evaluate their practice according to 50 Best Practice Statements in relation to: the crisis service; rapid assessment and intervention; and service resources. The second is a Best Practice Toolkit, consisting of resources for teams to develop specific aspects of their practice. The Resource Kit is available as a password-protected online resource. The research team will provide initial training on completion of the Fidelity Measure and use of the Best Practice Toolkit. Each TMCD in the intervention arm will complete the Fidelity Measure before the intervention phase, to determine areas where practice could be improved. The TMCD will then implement at least four relevant templates from the Best Practice Toolkit, during the implementation phase of 6 months. The Fidelity Measure will be repeated at the end of the intervention phase (after 6 months). Further details about the intervention are provided in Fig. 2.

#### Treatment as usual

TMCDs in the control arm will not have access to the Resource Kit, will not complete the Fidelity Measure, and will not use elements of the Best Practice Toolkit during the 6-month implementation phase of the trial, but will continue to conduct TAU. Both intervention group and TAU group are otherwise free to undertake all usual interventions.

#### Adherence

Adherence to the overall study protocol by teams in both arms of the study will be supported through weekly contact with the research team throughout the study. Following training in the use of the Resource Kit, practitioners in the intervention arm will complete a self-administered assessment to inform the research team of their level of understanding of the intervention. Subsequent adherence to the intervention will be adjudged based upon the use of the Resource Kit by TMCDs in the intervention arm. Specifically, adherence constitutes: the application and reporting of a Fidelity Measure score at both baseline and follow up time points; uptake and ongoing utilisation of at least four templates from the Best Practice Toolkit; and participation in weekly contact with the research team for the study duration.

### Setting

The study will take place in NHS Trusts across England which provide mental health services to older people and aims to recruit 30 TMCDs. To be eligible, participating Trusts must provide mental health crisis services for older people and must confirm that they can report all psychiatric hospital admissions, for people with dementia, and provide precise details of local acute NHS Trust(s) which admit people with dementia from the TMCD’s geographical catchment area, as defined by postcode. All NHS Trusts must also confirm, in advance of trial commencement, that the Resource Kit can be used by TMCDs during the intervention, regardless of the trial arm (intervention or control) to which the team is subsequently allocated.

### Participants

#### Inclusion criteria

Participating teams must be managing dementia mental health crises, defined as providing urgent mental health assessment and intervention for people with dementia in the community. Participants will be staff members of the TMCDs and new referrals of people with dementia and their carers supported by these teams.

#### Exclusion criteria

To avoid contamination between intervention and control groups, teams will be excluded if they share: immediate management; administrative or core clinical staff; or the same office as another team in the study. Teams in services undergoing a major service reorganisation during the study period or in the near future will also be excluded. If a team leader who has been exposed to the intervention becomes lead for a team in the control arm of the trial, the latter team will be excluded.

### Recruitment

#### Sites

NHS Trust sites will be recruited via professional and research networks across England, derived from earlier stages of the research programme. Participating Trusts will identify teams for inclusion in the study. Purposive recruitment will seek a diverse range of team service models and service user demographics, reflecting the earlier survey work undertaken in the study [12]. Each Trust shall confirm their capacity to undertake the trial by completing the Health Research Authority Statement of Activities, constituting a formal agreement with the study sponsor.

#### TMCDs (*n*=30) and practitioners (*n*=180)

In each team an Individual Team Manager, or a delegated senior practitioner, will be briefed by the research team about the trial and provided with participant information sheets. Two practitioners in each team will be sought to act as volunteer research coordinators and allowed up to 3 days from receiving the study information to decide if they wish to participate. These staff will recruit the remaining practitioners from the team.

#### People with dementia and their carers (*n*=450)

People with dementia and their carers will be identified from new referrals to the team’s caseload. Team practitioners will approach them and explain that their team is participating in the trial and that they are also invited to do so.

#### Consent process

During a site set-up visit to each TMCD the AQUEDUCT research team will take consent from the two TMCD practitioners acting as research coordinators. These research coordinators will arrange and confirm consent with their fellow TMCD practitioners, following the procedure used for confirmation of their own consent. This delegation of responsibility will be recorded formally in the Site Delegation Log.

People with dementia and carers will be approached by TMCD practitioners and given an information sheet, opportunities to ask questions and up to 3 days to decide if they wish to participate. If they agree, formal consent will be obtained from the person with dementia. This consent will be seen as a continuing process and at each meeting, the practitioner will determine their capacity (according to the Mental Capacity Act 2005 [24]) to give informed consent to take part in the research. A consent form will be completed separately with the carer regarding their involvement in the trial.

All participants will be informed of their right to withdraw from the research for any reason and at any time and that this decision will not impact on their current or future work within clinical services or access to and use of services. In certain circumstances, the CI may withdraw people with dementia from the trial if it is no longer considered to be in their best interest to continue.

### Outcome measures

**Fig. 3:**
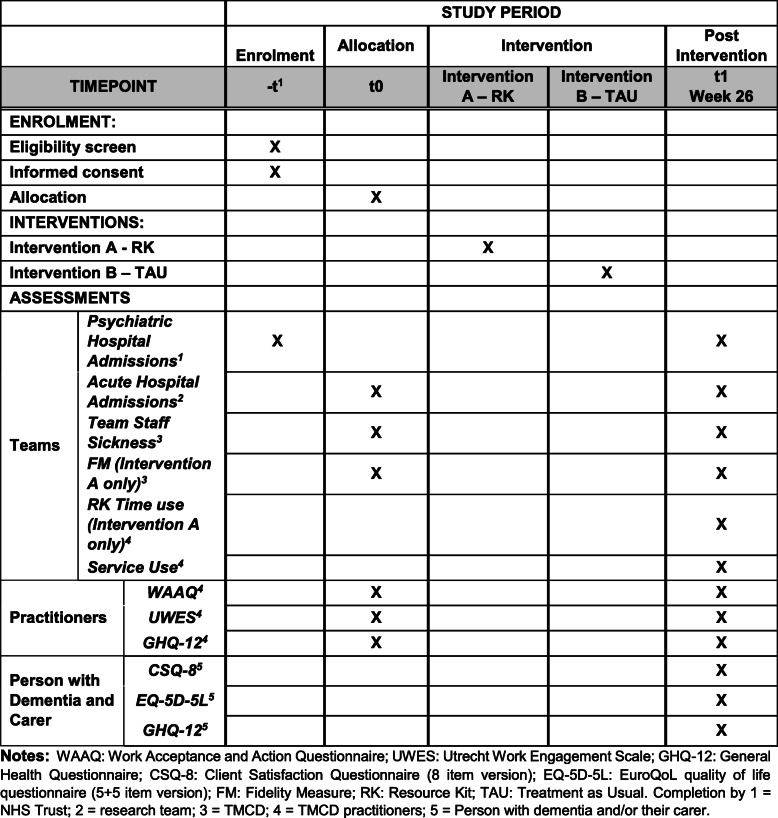
Schedule of enrolment, interventions, and assessment points

The schedule of outcome measures and assessment points is provided in the SPIRIT chart (Fig. 3).

#### Primary outcome measure

##### Psychiatric hospital admissions for people with dementia

All psychiatric hospital admissions for people with dementia from the TMCD catchment area (as defined by post codes) will be collated and reported at baseline and 6-month follow-up point for the preceding 6-month period.

#### Secondary outcome measures for people with dementia

##### Acute/general hospital admissions for people with dementia

All acute/general hospital admissions for people with dementia from the TMCD catchment area (as defined by post codes) will be collated and reported at baseline and 6-month follow-up point for the preceding 6-month period.

#### Secondary outcome measures for people with dementia and their carers

##### Service satisfaction (one measure)

The Client Satisfaction Questionnaire (CSQ-8) is a brief satisfaction with service measure and consists of eight items designed to measure satisfaction with health and human services in a single scale [25].

##### Quality of life and psychological wellbeing (two measures)

The EuroQol 5-dimension 5 level questionnaire (EQ-5D-5 L) is a brief assessment of the quality of life which comprises five dimensions: mobility, self-care, usual activities, pain/discomfort and anxiety/depression each rated by five levels of severity [26, 27]. The General Health Questionnaire (GHQ-12) is a brief 12-item scale which measures current mental health and psychological well-being [28].

#### Secondary outcome measures for TMCD practitioners

##### Work environment (two measures)

The Work Acceptance and Action Questionnaire (WAAQ) assesses psychological flexibility in relation to the workplace and comprises seven items which rate ability or willingness to remain engaged in occupational work while experiencing distressing thoughts and emotions [29]. The Utrecht Work Engagement Scale (UWES) is a 17-item scale measuring three dimensions of work engagement (vigour, dedication and absorption) [30, 31].

##### Wellbeing in work (two measures)

The General Health Questionnaire (GHQ-12) is a brief 12-item scale which measures current mental health and psychological well-being [28]. Sickness absence is used as a common proxy for work wellbeing and will be collated for all TMCD practitioners for each team.

##### Fidelity Measure (one measure)

The Fidelity Measure is a measure of practice quality in managing crises and part of the Resource Kit developed from the AQUEDUCT research programme. It will be completed by each TMCD in the intervention arm only.

#### Cost measures

##### Resource use (five measures)

Psychiatric hospital admissions for people with dementia within the TMCD’s catchment area (as defined by post codes) will be collated and reported at baseline and 6-month follow-up point for the preceding 6-month period. Acute/general hospital admissions for people with dementia within the TMCD’s catchment area (as defined by post codes) will be collated and reported at baseline and 6-month follow-up point for the preceding 6-month period. A record of both the number of permanent care home admissions and respite care admissions from each TMCD during the intervention phase will be collated to indicate use of other high-cost resources. In the intervention arm only, staff time use in implementing the Resource Kit and support time given to staff for this will capture additional costs of the intervention. Cost information will be collected using a ‘reduced list’ approach to costing [32], reflecting the necessary parsimonious adaptation of methodology required following the COVID-19 pandemic. This will avoid the use of expensive-to-collect items of data which are unlikely to vary and affect overall cost differences between the intervention arm and TAU arm, such as GP costs.

#### Other data collected

##### Serious adverse events

In accordance with Good Clinical Practice all data collectors will report deaths and adverse events that are life threatening, require or extend hospitalisation, result in disability or incapacity or are otherwise significant. All adverse events will be recorded in the case report form.

### Procedure

Overall involvement in the trial will be for 8 months, to include: provision of hospital admission data from all NHS Trusts at baseline and 6 months follow-up; initial set-up; completion of the Fidelity Measure before and after implementation of the Resource Kit; and delivery of all participant-completed measures. TMCDs in the intervention arm will implement the Resource Kit for 6 months and TMCDs in the control arm will deliver TAU for 6 months.

#### Data collection

Data will be collected from NHS Trusts, TMCDs, team practitioners, and new referrals of people with dementia and carers to these TMCDs.

Psychiatric hospital admissions data in the TMCD catchment area will be collated and reported by the relevant NHS Trust Department retrospectively at baseline and at 6-month follow-up. Acute/general hospital admissions data from each TMCD catchment area will be collected from each hospital by the research team retrospectively at baseline and at 6-month follow-up. Each TMCD in the intervention arm will complete the Resource Kit Fidelity Measure at baseline and 6-month follow up to identify gaps in their practice.

TMCD practitioners in both arms of the study will complete three questionnaires (WAAQ; UWES; GHQ-12) at baseline and at 6-month follow-up point. Sickness absence will be collated at team level for all TMCD practitioners at baseline and at 6-month follow-up point for the preceding 6-month periods. In both study arms, practitioners will record the number of permanent care home admissions and respite care admissions throughout the intervention phase for those people with whom the TMCD has engaged. TMCD practitioners in the intervention arm only will keep activity records to monitor time spent implementing the Resource Kit during the research.

After discharge from the TMCD service, people with dementia and carers will be asked to complete three questionnaires (CSQ-8, EQ-5D-5 L and GHQ-12). All measures can be completed within 6 weeks of discharge from the TMCD.

#### Random allocation

Following consent, each TMCD will be entered onto a web-based randomisation system and be randomly assigned to one of two arms, either using the Resource Kit or TAU with equal opportunity. The allocation will be determined by a computer-generated pseudo-random code using random permuted blocks of varying size, minimised by the number of people with dementia admitted to psychiatric hospital in the previous 6 months (either classified as low if the number is less than 25, or high if 25 or more) in each TMCD catchment area. The block size will not be disclosed to the research team. The randomisation system was set up and managed by the Research and Innovation team at the University of Nottingham (separate from the research team) in accordance with their standard operating procedure and is held on a secure server. The randomisation process is described in detail in a separate file and held securely within the statistics master file.

### Masking

People with dementia, carers, statisticians, and the DMC will be blinded to TMCD arm allocation until the data analysis is completed. However, masking of TMCDs is not possible.

### Data management

Study data will be managed using REDCap electronic data capture tools (version 10.0.5 LTS) [33, 34] hosted at the University of Nottingham. All data will be treated in a confidential manner and in accordance with UK data protection legislation. All research staff and practitioners involved in the study will be appropriately trained and supported with regards to the collection, storage, processing and disclosure of personal information. Unmasked researchers will enter data into REDCap software in accordance with study-specific guidance. Each participant will be assigned a unique identification code that will be used for all research data linkage and storage systems to ensure anonymity. Participants’ identifiable information will be stored securely, separate from the anonymised research data. A full audit trail will be maintained by recording all amendments to data specifying reason and time. Personal data will only be accessible to the minimum number of individuals necessary for data analysis or audit. Data may be inspected and audited by the sponsor and research ethics committee. Study investigators will have access to the study data by request from the CI and only anonymised data will be transferred to co-investigators at other sites. Following completion of the study non-personal data will be stored for five years in accordance with the terms of the Sponsor’s contract with the funder and personal data will be stored for three to 6 months to allow completion of the research, and for results to be disseminated to participants if required.

### Sample size

Information collected in different stages of the AQUEDUCT research programme, showed the average hospital admission count per TMCD catchment area over a 6-month period to be between 17 and 33. Following stakeholder consultation, a 20% reduction represented the minimum clinically significant difference in outcome. Therefore, 15 TMCDs will be required in each of the two study arms (30 in total) to detect a 7-point mean admission count difference between arms with 90% power at a two-tailed 0.05 significance level, assuming the count of hospital admissions follows a Poisson distribution. Stata 16 software was used for this power analysis. It is anticipated that no TMCD will withdraw from the study and that NHS Trusts will provide the required hospital admissions data for each TMCD; thus, the number of TMCDs required is unlikely to be influenced by lost information due to withdrawal. However, to manage risk of loss, additional team sites will be kept in reserve.

### Statistical analysis

The data analysis will be conducted on an intention to treat basis. The treatment effects estimate (95% CI) on the primary outcome and the number of general hospital admissions will be quantified using Poisson regression with binary arm status as an exploratory variable and the size of the population with dementia within each TMCD catchment area as an offset. An over-dispersion check will be performed, and a negative binomial regression model will be used if there is evidence that the outcome variance is greater than the mean. The treatment effect estimates on TMCD practitioners’ and people with dementia and carers’ outcome measures will be explored using multilevel modelling with the TMCD as a level two analytical unit and baseline measures included as covariates [35, 36]. Skewed continuous outcome variables will be transformed for multilevel modelling, and nonlinear multilevel modelling will be performed for categorical outcomes. No interim analysis is planned, and safety and adverse event information will be presented descriptively. Missing values will be imputed via analytical modelling assuming missing at random status. Stata 17 will be used to analyse the data.

#### Economic evaluation

A cost-consequences analysis will be undertaken, laying out a specified list of costs, including Resource Kit costs, service use and trial outcomes (hospital admissions and quality of life measures). Bootstrapping will be used to generate incremental cost-effectiveness ratios and plot cost-effectiveness acceptability curves. A cost-effectiveness analysis will be undertaken, examining Resource Kit costs and secondary care use costs (using national unit cost measures) and comparing these with changes in the primary outcome measure. Sensitivity analyses will be conducted to assess the implications of variations in team staff case-mix upon outcomes.

The primary economic evaluation will be a cost-utility analysis from an NHS perspective based on the within-trial period. The incremental cost per QALY (quality-adjusted life year) gained for the Resource Kit versus TAU will be calculated for the average TMCD. QALYs gained will be estimated as the number of weeks multiplied by the utility of observed survival for patients and carers within the average TMCD. The utility values will be estimated from the EQ-5D-5 L health status questionnaire [27], completed at follow-up and the associated published societal utility tariffs.

An economic analysis plan will be approved by the DMC before data are accessed. Statistical methods, such as accounting for missing data and adjustment will be consistent with the statistical analysis plan.

### Qualitative aspects of trial

To enhance understanding of participants’ experience of and engagement with the intervention the trial includes a qualitative component. Study-specific questionnaires will be completed by 45 TMCD practitioners from the intervention arm of the trial (three practitioners per team), and researchers will conduct semi-structured interviews remotely, via telephone or multimedia, with 12 people with dementia or their carers who received input within the previous 6 weeks from a TMCD in the intervention arm.

Both thematic and narrative analysis of interview transcripts will be deployed to identify experiences both of staff providing the intervention and of people with dementia and their carers receiving it. At least two researchers will review each transcript to increase trustworthiness. Once all aspects are coded, key themes and processes will be identified through a process of conceptual abstraction. The point of reaching thematic saturation [37] will be agreed with members of the wider research team.

## Discussion

The AQUEDUCT programme is designed to address the better management of crises in the care of people with dementia. The trial is built upon knowledge gaps identified in previous work by team members [16, 19] and has involved literature reviews [12], the development of practice modules for service improvement [14, 20], and a feasibility study [23].

There is limited evidence for the precise content of crisis resolution services for people with dementia and their effectiveness and cost-effectiveness. Nonetheless, people with dementia and carers value a coordinated and well-focused approach to addressing crises in their care [15]. Policy in dementia care continues to emphasise the importance of care at home and of avoiding unnecessary admissions to hospital care [3–5, 38]. There is therefore a need for more precise evidence of the form and content of an improved model of crisis services for people with dementia and their carers.

Health and social care services need to better understand what interventions prevent hospitalisation and enhance well-being for people with dementia and carers in the period leading up to or during a crisis. This trial can contribute to such a knowledge base if the intervention proves to be clinically and cost-effective. It is hypothesised that a more precise model of crisis management, supported through provision of a Resource Kit, can reduce admissions to hospital and improve the well-being of people with dementia and their carers.

This study could have wide-ranging value in improving crisis management for people with dementia and their carers and evidencing what works for service commissioners and planners, health and social care professionals, and researchers both nationally and internationally. Evidence from this study will be submitted for publication in peer-reviewed journals and findings presented at relevant conferences and events. To achieve impact, the Resource Kit will be made available after the trial has ended, as part of our programme of implementation and dissemination.

### Trial status

The trial is due to commence recruitment in July 2021 and will continue until November 2022.

## Supplementary Information

**Additional file 1.** Spirit checklist.

## Data Availability

The study datasets are available from the CI on reasonable request.

## Abbreviations

AQUEDUCT: Achieving Quality and Effectiveness in Dementia Using Crisis Teams
CI: Chief Investigator (Professor Martin Orrell)
CONSORT: Consolidated Standards of Reporting Trials
COVID-19: (Novel) Coronavirus disease 2019 (WHO nomenclature)
CSQ-8: Client Satisfaction Questionnaire – 8 item version
DMC: Data Monitoring Committee
EQ-5D-5 L: EuroQoL quality of life questionnaire – 5 + 5 item version
GHQ-12: General Health Questionnaire – 12 item version
NHS: National Health Service
NIHR: National Institute for Health Research
PPI: Patient and public involvement
QALY: Quality-adjusted life year
REDCap: Research Electronic Data Capture
SPIRIT: Standard Protocol Items: Recommendations for Intervention Trials
TAU: Treatment as usual
TMCD: Team Managing Crisis in Dementia
TMCDs: Teams Managing Crisis in Dementia
TMG: Trial Management Group
TSG: Trial Steering Group
UWES: Utrecht Work Engagement Scale
WAAQ: Work Acceptance and Action Questionnaire
